# Activation of the P62-Keap1-NRF2 Pathway Protects against Ferroptosis in Radiation-Induced Lung Injury

**DOI:** 10.1155/2022/8973509

**Published:** 2022-07-05

**Authors:** Xuan Li, Jingyao Chen, Sujuan Yuan, Xibing Zhuang, Tiankui Qiao

**Affiliations:** ^1^Jinshan Hospital Center for Tumor Diagnosis & Therapy, Jinshan Hospital, Fudan University Shanghai Medical School, Shanghai, China; ^2^Fudan University Shanghai Medical School, Shanghai, China

## Abstract

Radiation-induced lung injury (RILI) is one of the most common, serious, and dose-limiting toxicities of thoracic radiotherapy. A primary cause for this is the radiation-induced cell death. Ferroptosis is a recently recognized form of regulated cell death, characterized by the accumulation of lipid peroxidation products and lethal reactive oxygen species (ROS). The ROS generated by irradiation might be the original trigger of ferroptosis in RILI. In addition, activation of the P62-Kelch-like ECH-associated protein 1 (Keap1)-nuclear factor erythroid 2-related factor 2 (NRF2) pathway has been shown to blunt ferroptosis and thus acts as a protective factor. Therefore, this study aimed to explore the protective effect of the P62-Keap1-NRF2 pathway against radiation-induced ferroptosis in alveolar epithelial cells. First, we found that radiation induced ferroptosis in vitro using a RILI cell model, which could be significantly reduced by ferrostatin-1 (Fer-1), a specific ferroptosis inhibitor. Additionally, overexpression of P62 interacted with Keap1 to facilitate the translocation of NRF2 into the nucleus and promote the expression of its target proteins, including quinone oxidoreductase 1 (NQO1), heme oxygenase 1 (HO1), and ferritin heavy chain 1 (FTH1). In summary, our results demonstrated that the activation of the P62-Keap1-NRF2 pathway prevents radiation-induced ferroptosis in RILI cells, providing a theoretical basis of finding a potential therapeutic approach for RILI.

## 1. Background

In addition to chemotherapy and surgery, radiotherapy is the main modality used to treat cancer. However, radiation-induced lung injury (RILI) is a potentially fatal complication of thoracic radiotherapy, consisting of radiation-induced pneumonitis (RIP) and radiation-induced lung fibrosis (RILF) [1]. About 5-20% of patients suffer from RILI, which severely limits the curative effects of radiation therapy and diminishes the quality of life of patients [2]. A better understanding of the precise molecular mechanism of RILI is crucial to developing new strategies to minimize it.

Ferroptosis has recently been identified as a form of regulated cell death (RCD) distinct from apoptosis, triggered by the accumulation of lipid peroxides as the lethal event due to decreased degradation by glutathione peroxidase [3, 4]. Ferroptosis is associated with multiple disorders, such as acute kidney injury, Parkinson's disease, carcinoma, stroke, intracerebral hemorrhage, traumatic brain injury, and ischemia-reperfusion injury [5–7]. During ferroptosis, iron accumulation and lipid peroxidation are key signals that initiate membrane oxidative damage [8]. Ferroptosis results from abnormal iron metabolism in either of two ways. One is mediated by iron-mediated reactive oxygen species (ROS) produced by the Fenton reaction, and the other is involved in the activation of iron-containing enzymes [9].

P62 is a ubiquitin-binding autophagy receptor and signaling protein, triggering a self-amplifying autoregulatory loop that sustains nuclear factor erythroid 2-related factor 2 (NRF2) activation [10]. Both NRF2 and the Kelch-like ECH-associated protein 1 (Keap1) play an essential role in antioxidant responses [11]. An electrophile or an oxidant can modify Keap1, causing conformational changes which stabilize the Keap1-NRF2 interaction, preventing NRF2 proteasomal degradation [12]. What is more, NRF2 plays a pivotal role in attenuating inflammation and combating oxidative stress [13, 14].

The increasing amount of evidence indicates that the generation of ROS from radiation is the primary factor contributing to RILI, and that activating the P62-Keap1-NRF2 pathway can inhibit the occurrence of ferroptosis [15]. Based on our previous research, ferroptosis was implicated in RILI in mice and the NRF2 signal pathway-regulated ferroptosis [16]. Yet, the underlying mechanism of ferroptosis in RILI, including the interaction between ferroptosis-related genes, remains poorly understood.

Therefore, in this study, we developed a RILI cell model in which alveolar epithelial cells were exposed to 10Gy and explored the critical signal transduction pathways involved in ferroptosis induction in RILI. We demonstrated that radiation-induced oxidative stress inhibited ferroptosis by activating the P62-Keap1-NRF2 pathway in RILI cells. Furthermore, upregulation of NRF2 protected the RILI cells against ferroptosis by upregulating several antioxidant proteins that participate in iron and ROS metabolism, including quinone oxidoreductase 1 (NQO1), heme oxygenase 1 (HO1), and ferritin heavy chain 1 (FTH1). Together, our results revealed a novel therapeutic strategy for preventing and managing RILI.

## 2. Materials and Methods

### 2.1. Cell Culture and Reagent

The human NSCLC cell line A549 (Cat. No: KG007) was obtained from the Cell Bank of the Chinese Academy of Sciences (Shanghai, China). The cells were cultured in RPMI-1640 medium (Keygentec, China) containing 10% fetal bovine serum (CellSera, Australia), incubated at 37°C in an environment of 5% CO2 and 95% humidity. Ferrostatin-1 (MCE, USA), the first specific inhibitor of ferroptosis with clear inhibition effects and good specificity, was manufactured to the proper concentration according to the product's instructions.

### 2.2. Experimental Design

A549 cells were randomly divided into ten groups with different treatments as follows: (1) control group, (2) irradiation (IR) group, (3) IR + ferrostatin-1(Fer-1) group, (4) IR + LV-NRF2 group, (5) IR + LV-P62 group, (6) IR + siRNA-Keap1 group, (7) LV-NRF2 group, (8) LV-P62 group, (9) siRNA-Keap1 group, and (10) Fer-1 group. During irradiation, the cells were exposed to dose of 0, 2, 4, 6, 8, and 10 Gy X-ray radiation. The beam used was a 6 MV X-ray at a dose rate of 2 Gy/min. In IR + Fer-1 group, cells were treated with different concentration of Fer-1 30 min before radiation.

### 2.3. Clonogenic Assay

For standard clonogenic assays, A549 cells were seeded into 6-well plates at density of 2000 cells per well and incubated for 24 h. Then, the cells were pretreated with Fer-1 for 30 min, followed by 0, 2, 4, 6, 8, and 10 Gy X-ray radiation. The cells were incubated at 37°C for another 12 days to allow for colony formation, followed by crystal violet staining. Only viable colonies consisting of 50 or more cells were counted.

### 2.4. Cell Viability Assay

A549 cells were plated at the density of 5 × 10^3^ cells per well in 96-well plates. After overnight culture, cells were pretreated with various concentrations of Fer-1 for 30 min, followed by 0, 2, 4, 6, 8, and 10 Gy X-ray radiation. Cell viability was evaluated using Cell Counting Kit-8 Assay Kit (Do Jindo Laboratories, Kumamoto, Japan) according the manufacturer's instructions.

### 2.5. Quantitative Real-Time Polymerase Chain Reaction

Total RNA isolation was carried out using the RNA Purification Kit (Yi Shan Biotechnology Company, Shanghai, China). The complementary DNA (cDNA) was synthesized and amplified with the reverse transcription kit (Takara, Osaka, Japan). A quantitative real-time PCR kit (Takara, Osaka, Japan) was used to prepare cDNA from various cell samples, along with GAPDH as an internal control, and specific primers were used for amplification (NRF2: forward 5′-TCTGCCAACTACTCCCAGGT-3′ and reverse 5′- AATGTCTGCGCCAAAAGCTG -3′; P62: forward 5′-CCCTCTCCCAGATGCTGTCCAT-3′ and reverse 5′-G CCGCTCCGAT GTCATAGTTCT-3′; Keap1: forward 5′-CGTGGCTGTCCTCAATCGTCTC-3′ and reverse 5′-CGCTTCGGATGGTGTTCATTGC-3′; GAPDH: forward 5′-CAAATTCCATGGCACCGTCA-3′ and reverse 5′-AGCATCGCCCCACTTGATTT-3′).

### 2.6. Western Blot Analysis

Cells were lysed in RIPA buffer containing 1 nM phenylmethylsulfonyl fluoride (PMSF) and phosphatase and protease inhibitors on ice. Then, the lysates were centrifuged at 12,000 g for 20 min at 4°C, and the protein concentration of the supernatant was determined using the BCA Protein Assay Kit (Thermo Scientific, USA). Equal amounts of protein were separated via 8% to 10% SDS-PAGE and transferred to polyvinylidene fluoride (PVDF) membranes (Millipore, Bedford, MA, USA). After blocking with 5% skim milk for 1 h at room temperature, membranes were incubated with primary antibodies overnight at 4°C, followed by horseradish peroxidase-coupled secondary antibodies incubation. The details of primary antibodies used in this research were shown as follows: P62 (1 : 10000, ab155282, Abcam, UK), Keap1 (1 : 500, 10503-2-AP, Sanying BioTECH, China), Nrf2 (1 : 1000, ab62352, Abcam, UK), HO1 (1 : 500, 10701-1-AP, Sanying BioTECH, China), NQO1 (1 : 500, 11451-1-AP, Sanying BioTECH, China), FTH1 (1 : 1000, ab170888, Abcam, UK), and rabbit anti-GAPDH (1 : 10000, KGAA002, KeyGEN BioTECH, China). The immunocomplexes were subsequently detected on photographic film using a chemiluminescence reagent (Millipore, Bedford, MA, USA).

### 2.7. Morphological Observation of Mitochondria and Mitochondrial Membrane Potential Assay

The mitochondria morphology of A549 cells was observed by transmission electron microscopy (TEM). A549 cells were fixed with 2.5% glutaraldehyde in phosphoric acid buffer, postfixed in 1% osmium acid for 2 h at room temperature, and dehydrated in a graded series of ethanol. The ultrathin sections were cut with a diamond knife and stained with 3% uranium acetate and lead citrate. Capture the TEM images using transmission electron microscope (JEM-1011, Japan).

An assay kit for measuring mitochondrial membrane potential with JC-1 (KeyGEN BioTECH, Nanjing, China) was used. Briefly, cells were stained with JC-1 working solution for 20 min at 37°C, washed twice with incubation buffer, and then analyzed by flow cytometry (Becton-Dickinson, USA).

### 2.8. Measurement of Intracellular Fe^2+^ and ROS

The intracellular Fe^2+^ was determined using the fluorescent indicator PGSK (KeyGEN Biotech, Nanjing, China). Cell suspensions were collected and supplemented using the PGSK probe. After 30 min of incubation, cells were centrifuged, washed, and resuspended. Then, the relative PGSK level was measured by flow cytometry (Becton-Dickinson, USA).

The ROS level was detected with the fluorescent probe DCFH-DA (KeyGEN BioTECH, Nanjing, China) by flow cytometry. Briefly, A549 cells with different treatments were incubated with DCFH-DA for 30 min at 37°C and subsequently washed with PBS. After incubation, the fluorescence of the cells was measured using flow cytometry (Becton-Dickinson, USA).

### 2.9. Measurement of Lipid Peroxidation and Glutathione

Malondialdehyde (MDA) content, a product of lipid peroxidation, was used to assess the level of lipid peroxidation. The relative MDA concentration in cell lysates was assessed using a Lipid Peroxidation (MDA) Assay Kit according the manufacturer's instructions. Briefly, the MDA in the sample reacts with thiobarbituric acid (TBA) to generate an MDA-TBA adduct, which can be easily quantified colorimetrically (OD = 532 nm). Values were normalized for protein content of the lysates. The relative glutathione (GSH) concentration in cell lysates was estimated using a Glutathione Assay Kit according to the manufacturer's instructions.

### 2.10. Immunofluorescence

After treatment, cells were plated on glass coverslips and fixed with 4% paraformaldehyde for 15 minutes and then blocked with 5% BSA for 1 hour at room temperature. Immunofluorescence was performed by incubating with antibodies against GPX4 (1 : 200, ab125066, Abcam, UK) and ACSL4 (1 : 100, ab155282, Abcam, UK) at 4°C overnight. On the second day, cells were incubated with Goat Anti-Rabbit IgG antibodies and washed thrice in PBS. Nuclei were counterstained using 4′,6-diamidino-2-phenylindole (DAPI) for 10 minutes. The cells were observed under a fluorescence microscope.

### 2.11. Statistical Analysis

Statistical analysis was performed through GraphPad Prism 7 (GraphPad Software, San Diego, CA, USA). Three samples of each group were used to calculate the data. Results were presented as the mean ± SD. The differences between pairwise comparisons were determined using Student's *t*-test. The comparisons among multiple groups were carried out by one-way ANOVA, followed by a Bonferroni correction. *P* values of less than 0.05 were considered statistically significant.

## 3. Results

### 3.1. Radiation-Induced Ferroptosis in A549 Cells

**Figure 1:**
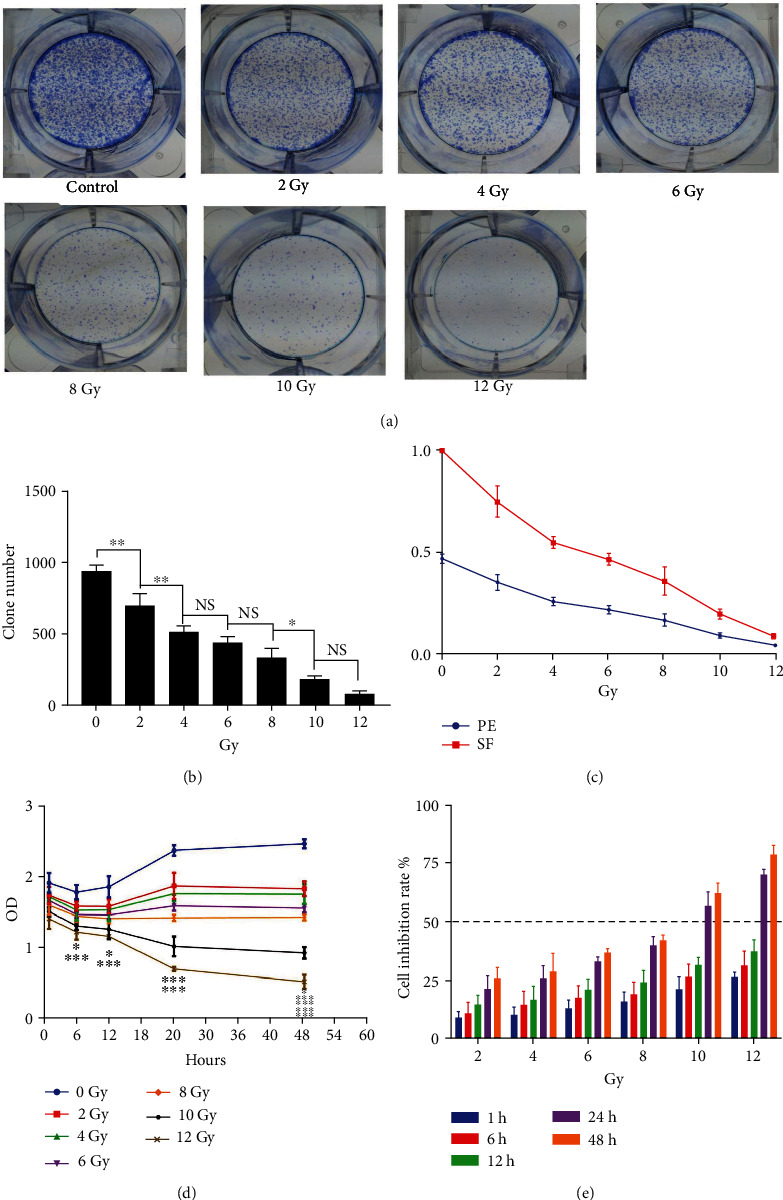
RILI cell model. (a, b) Changes in colony formation number of A549 cells in six-well plate under different radiation doses. (c) Cell clone formation rate (PE) and cell survival fraction (SF) under different radiation doses. (d) OD values at different time points after irradiation in different radiation dose groups using CCK-8 test. (e) Cell inhibition rate in different dose groups at different time points after irradiation (data shown as mean ± SD, one-way ANOVA followed by a Bonferroni correction, NS represented no statistical difference. ∗*P* < 0.05, ∗∗*P* < 0.01, and ∗∗∗*P* < 0.001).

A549 cells were used to establish RILI cell model in our study. In response to different doses of radiation and cell proliferation activity measured by cell clone formation and CCK-8 tests, the clone number and the cell viabilities of A549 cells decreased in a dose-dependent manner (Figure 1). The inhibition rate of cells exposed to 10Gy X-rays 24 h after irradiation (56.7%) was the closest to the fifty percent inhibitory concentration (IC50). Therefore, a 10Gy 6 MV X-ray irradiation and detection of RILI-related indexes 24 hours after irradiation is the best way to establish a RILI cell model.

**Figure 2:**
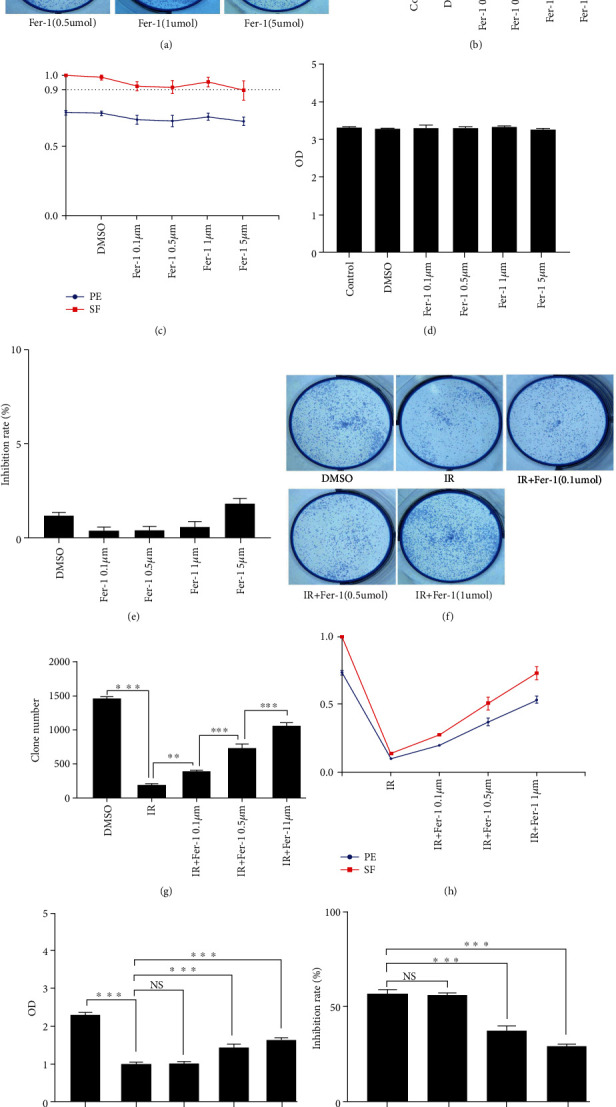
Cytotoxicity and effective concentration of Fer-1 on RILI cell. (a, b) Changes in colony formation number of A549 cells in six-well plate under different concentrations of Fer-1. (c) A549 cells clone formation rate (PE) and cell survival fraction (SF) under different concentrations of Fer-1. (d, e) OD values and cell inhibition rate of A549 cells in different concentrations of Fer-1 with CCK-8. (f, g) Changes of colony formation number of RILI cells in six-well plate under different concentrations of Fer-1. (h) RILI cells clone formation rate (PE) and cell survival fraction (SF) under different concentrations of Fer-1. (i, j) OD values and cell inhibition rate of RILI cells in different concentrations of Fer-1 using CCK-8 test (data shown as mean ± SD, one-way ANOVA followed by a Bonferroni correction, NS represented no statistical difference. ∗*P* < 0.05, ∗∗*P* < 0.01, and ∗∗∗*P* < 0.001).

Various concentrations (0.1, 0.5, 1, and 5uM) of Fer-1 did not exhibit significant cytotoxic effects on A549 cells as demonstrated by the cell clone formation and CCK-8 tests (Figure 2). We then administrated Fer-1 to RILI cells at concentrations of 0.1, 0.5, and 1uM and found that with an increase in Fer-1concentration, the cell clone number increased and the cell inhibition rate decreased. Overall, these data showed that Fer-1 suppressed radiation-mediated ferroptosis in RILI cells in a dose-dependent manner, and 1uM was selected as the effective dose in the follow-up study.

### 3.2. Ferroptosis Inhibitor Exerted a Protective Effect in Ferroptosis in RILI Cells

**Figure 3:**
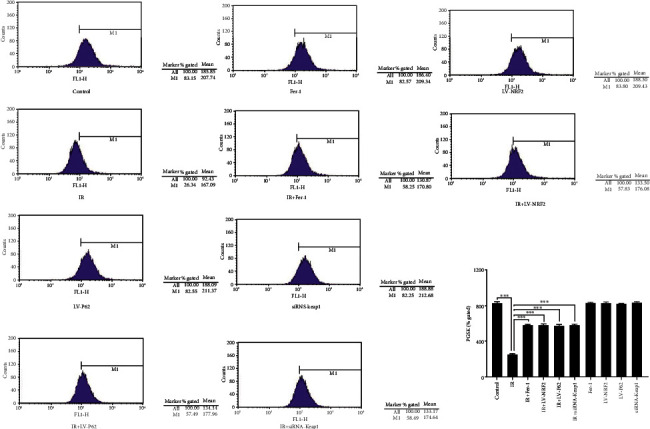
The intracellular Fe^2+^ with the fluorescent indicator PGSK (data shown as mean ± SD, one-way ANOVA followed by a Bonferroni correction, ∗∗∗*P* < 0.001).

Studies have shown that ferroptosis is characterized by redox imbalance and impaired lipid metabolism [17, 18]. To further verify the availability of RILI cell model and the role of ferroptosis in RILI, the intracellular Fe^2+^ was determined using the fluorescent indicator PGSK (Figure 3). As compared with the control group, Fe^2+^ was dramatically reduced in the IR group, while Fer-1 significantly reversed the decrease. The same protective effect of Fer-1 was achieved by overexpressing P62 or NRF2.

**Figure 4:**
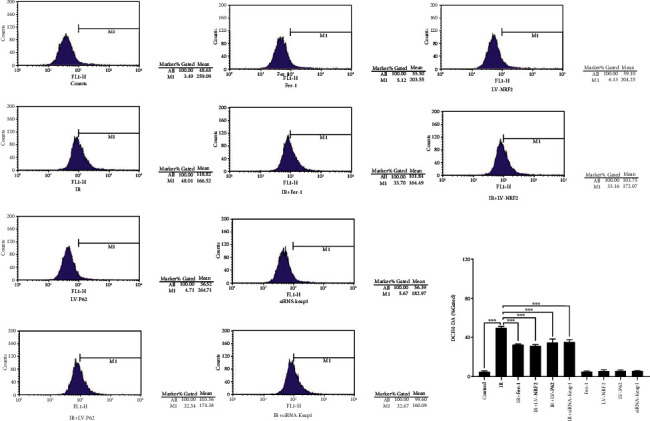
The ROS levels with the fluorescent probe DCFH-DA (data shown as mean ± SD, one-way ANOVA followed by a Bonferroni correction, ∗∗∗*P* < 0.001).

The abnormal increase in ROS tends to reflect RILI [2]. We then detected the ROS level using the fluorescent probe DCFH-DA by flow cytometry. As shown in Figure 4, the ROS level after radiation was obviously higher than that without radiation. Likewise, Fer-1, silencing of Keap1, or overexpressing of P62 or NRF2 improved the ROS level, confirming the contribution of ferroptosis to RILI.

**Figure 5:**
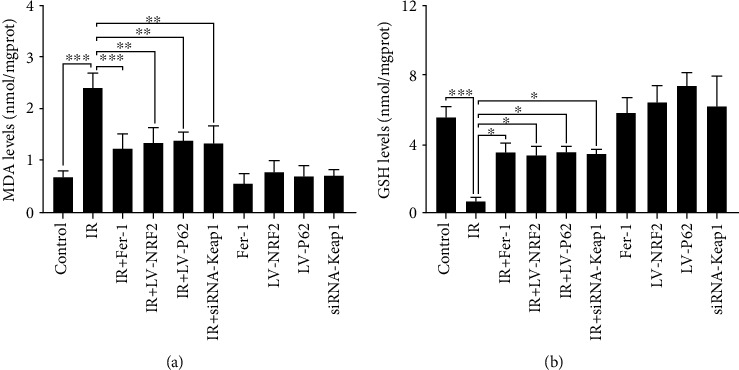
Measurement of lipid peroxidation and glutathione. (a, b) MDA and GSH levels of each group (data shown as mean ± SD, one-way ANOVA followed by a Bonferroni correction, ∗*P* < 0.05, ∗∗*P* < 0.01, and ∗∗∗*P* < 0.001).

Oxidative stress caused by redox imbalance may produce a variety of lipid peroxidation products or alter lipid metabolism [19]. Oxidative stress was assessed by evaluating contents of MDA and GSH. The results showed that the irradiation group showed elevated contents of MDA and decreased GSH levels, while Fer-1, silencing of Keap1, or overexpression of P62 or NRF2 suppressed the increase of GSH depletion and contents of MDA remarkably (Figure 5).

As a result, ferroptosis inhibitors protected RILI cells from redox imbalance and impaired lipid metabolism, and the P62-Keap1-NRF2 pathway regulated ferroptosis in RILI.

### 3.3. Ferroptosis Inhibitor Protected Mitochondrial Membrane from Radiation-Induced Injury

**Figure 6:**
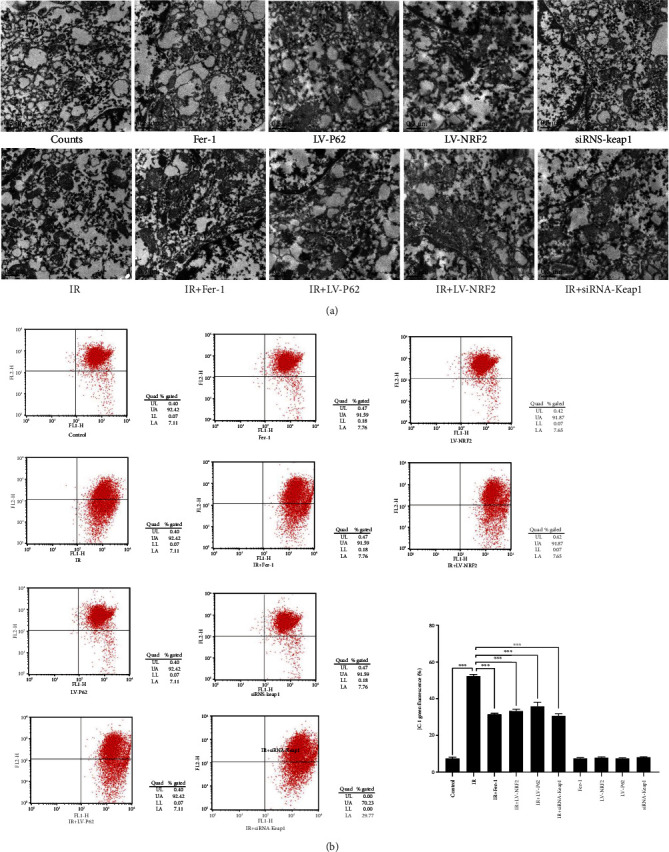
Changes in mitochondrial membrane of different groups. (a) The morphological changes in mitochondrial membrane of each group were observed by transmission electron microscope. Scale bar: 0.5 *μ*m. (b) Mitochondrial membrane potential in each group with JC-1 (data shown as mean ± SD, one-way ANOVA followed by a Bonferroni correction, ∗∗∗*P* < 0.001).

Studies have shown that ferroptosis is characterized by a decline in mitochondrial membrane potential, an obvious reduction in mitochondrial morphology, and an increase in membrane density [20, 21]. TEM results indicated that radiation decreased mitochondrial volume and increased mitochondrial membrane density [22]. As shown in Figure 6(a), shrinkage of mitochondrial morphology and the increase of membrane density were observed in IR group compared to the normal mitochondria in the groups with no irradiation. As compared with the IR group, we observed mitochondria morphology with larger volume in IR + Fer-1 group, IR + LV-NRF2 group, IR + LV-P62 group, and IR + siRNA-Keap1 group, and the membrane density of mitochondria decreased partly, especially in IR + LV-NRF2 group, IR + LV-P62 group, and IR + siRNA-Keap1 group. We then detected mitochondrial membrane potential by flow cytometry using the fluorescent probe JC-1 (Figure 6(b)). The mitochondrial of RILI cells were significantly depolarized. Fer-1 could ameliorate mitochondrial depolarization, further suggesting RILI involves ferroptosis. Likewise, silencing Keap1 or overexpression of P62 or NRF2 resulted in the reduced mitochondrial membrane potential.

### 3.4. P62-Keap1-NRF2 Pathway Had Been Activated in Ferroptosis in RILI Cells

**Figure 7:**
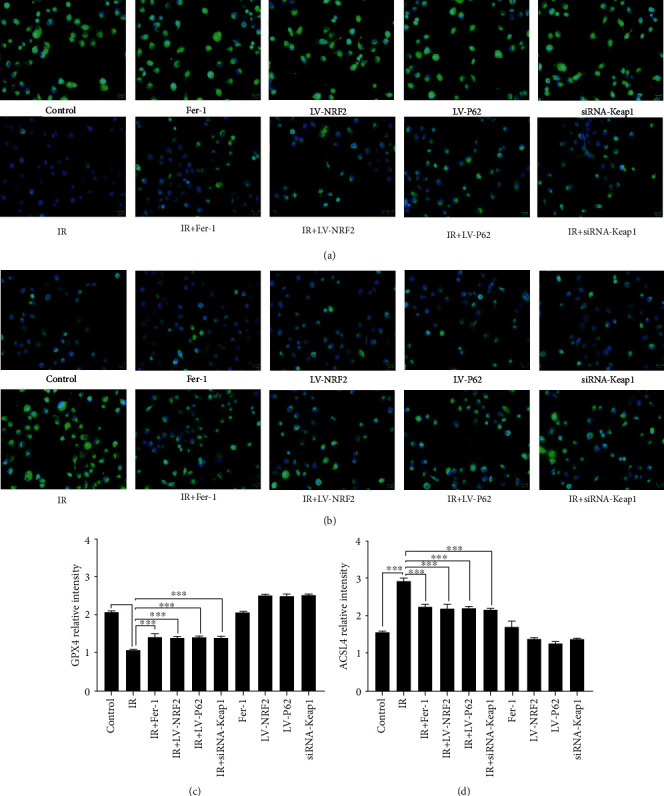
Expression levels of GPX4 and ACSL4 in cells of each group. (a, c) Representative fluorescence micrographs of GPX4 staining and quantification of GPX4 expressions of each group. (b, d) Representative fluorescence micrographs of ACSL4 staining and quantification of ACSL4 expressions of each group (data shown as mean ± SD, one-way ANOVA followed by a Bonferroni correction, ∗∗∗*P* < 0.001). Scale bar is 20 *μ*m.

The underlying mechanism of ferroptosis in RILI was determined by immunofluorescence analysis of the activation of ferroptosis markers (Figure 7). Results showed that IR group significantly decreased the expression of GPX4, a key regulator enzyme of ferroptosis, and increased the expression of ACSL4, another pivotal factor determining the sensitivity of ferroptosis.

**Figure 8:**
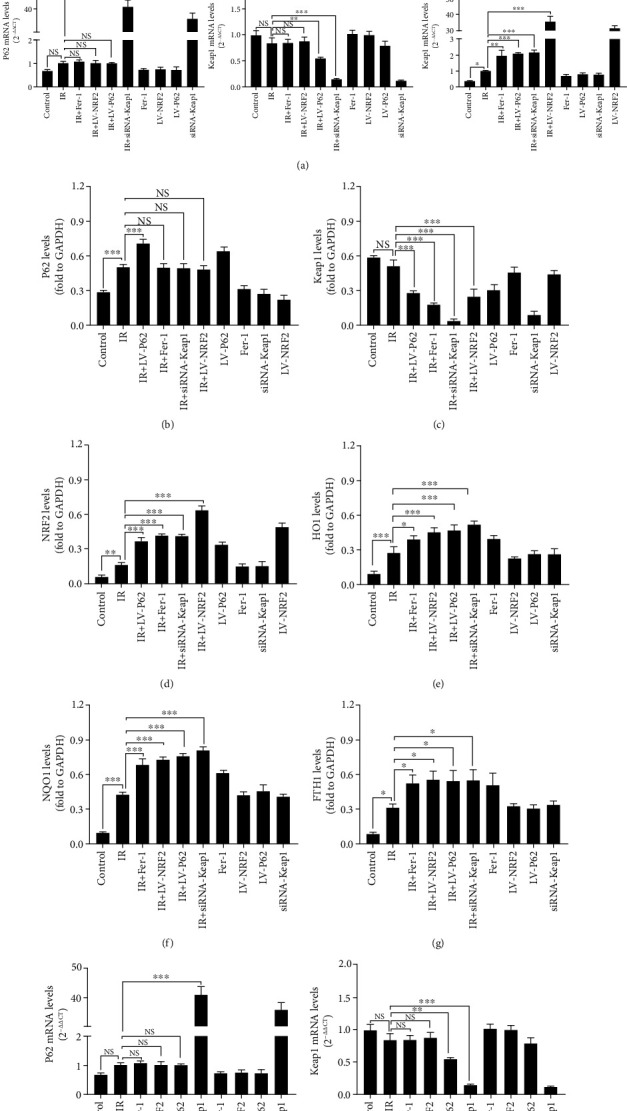
The changes in gene expression of P62-Keap1-NRF2 pathway in RILI cells. (a–g) The expressions and quantification of P62, Keap1, NRF2, HO1, NQO1, and FTH1 by western blotting. (h–j) The mRNA levels of P62, Keap1 and NRF2 were evaluated by real-time PCR (data shown as mean ± SD, one-way ANOVA followed by a Bonferroni correction, NS represented no statistical difference. ∗*P* < 0.05, ∗∗*P* < 0.01, and ∗∗∗*P* < 0.001).

Several studies have demonstrated that P62-Keap1-NRF2 pathway was involved in the regulation of ferroptosis [22, 23]. In order to further explore the role of the P62-Keap1-NRF2 pathway in RILI cells, siRNA Keap1, LV-P62, and LV-NRF2 constructs were transfected to silence Keap1 and overexpress P62 and NRF2. Following the transfection, a quantitative real-time PCR was performed to verify the efficiency of the transfection, indicating that the mRNA expression of P62 and NRF2 had increased significantly compared with the control and the LV-control groups. Overexpressing P62 decreased Keap1 levels and increased NRF2 levels as determined by western blotting and real-time PCR (Figure 8). Despite this, overexpressing NRF2 or silencing Keap1 did not influence the expression of P62, indicating that Keap1 and NRF2 are downstream of P62. Downregulation of Keap1 expression could promote NRF2 expression into nucleus. The downstream antioxidant proteins such as HO1, FTH1, and NQO1 were also detected. The results showed upregulation of P62, or NRF2 facilitated the expression of antioxidative protein such as HO1, FTH1, and NQO1. Fer-1 intervention did not induce the expression of P62 protein and mRNA in RILI cells, but it did remarkably induce Keap1 protein expression, but not its mRNA level. Both NRF2 protein and mRNA levels increased significantly, as did levels of antioxidant proteins HO1, FTH1, and NQO1. As a whole, the P62-Keap1-NRF2 pathway was responsible for the ferroptosis in RILI.

## 4. Discussion

Cell death is one of the critical pathophysiological processes in the occurrence and development of RILI [24]. Upon death of the alveolar epithelial cells, the alveolar respiratory membranes will become damaged, leading to a breakdown of the pulmonary surfactant, resulting in collapse of alveoli and subsequent pulmonary edema, and finally the occurrence of RILI [1, 25]. Therefore, it is representative to the construction of RILI cell model with alveolar epithelial cells, and thus, A549 cells were selected in our study. Through different doses of radiation and cell proliferation activity detected by cell clone formation and CCK-8 tests, we found that 10Gy 6 MV X-ray radiation and the detection of RILI-related indexes 24 hours after irradiation was the most effective way to establish RILI cell model. Fer-1 was the first specific inhibitor of ferroptosis, exhibiting obvious inhibition effect and great specificity, which was widely used in ferroptosis-related experiments [8, 26]. Based on cell clone formation and CCK-8 tests, we determined Fer-1 1uM inhibited the ferroptosis of RILI cells, consistent with the effective dose inhibiting ferroptosis in a variety of peroxidation diseases [27, 28].

To verify the availability of RILI cell models and the role of ferroptosis in RILI, we intervened RILI cells with Ferr-1, and then detected the associated indicators of RILI and ferroptosis. According to our findings, ROS and MDA levels in RILI cells increased significantly, and the levels of GSH decreased dramatically, suggesting a successful formulation of a RILI cell model. Furthermore, the reduction of ROS and MDA levels as well as the increase of GSH levels indirectly indicated ferroptosis in RILI.

The decline of mitochondrial membrane potential, the obvious shrinkage of mitochondrial morphology, and the increase of membrane density are typical signs of ferroptosis, as well as the direct morphological evidence for detecting ferroptosis [29]. The metabolism disorder of iron ions disturbed by intracellular peroxidation and the decrease of Fe^2+^ level are the metabolic characteristics of ferroptosis [8]. GPX4 is a key regulator enzyme of ferroptosis, and its expression level is usually used as an indicator of ferroptosis [30]. Furthermore, ACSL4 is another crucial factor for determining the sensitivity of ferroptosis in cells by enriching *ω*6 fatty acids, and increases in its expression can also serve as a marker of ferroptosis [31, 32]. Using electron microscopy, we observed that the mitochondrial morphology shrank, the membrane density thickened, Fe^2+^ and GPX4 levels decreased considerably, and ACSL4 expression increased in RILI cells, which were all improved after Fer-1 treatment.

The NRF2 signaling pathway is responsible for antioxidation in RILI, as well as regulating ferroptosis [33, 34]. Research has shown that the activation of the P62-Keap1-NRF2 pathway can inhibit ferroptosis [22, 23]. According to our previous study, NRF2 was involved in ferroptosis regulation in RILI, but its precise mechanism and how each part is interrelated remains unclear.

P62 plays a critical role in regulating various intracellular pathways and involves in apoptosis, autophagy, and other functions [35]. P62 is able to activate the expression of Keap1-NRF2 pathways, whereas P62 activates NRF2 in a positive feedback loop, and the P62-NRF2 pathway can also be activated even without the modification of Keap1 [36–38]. NRF2 plays a key role in the regulation of endogenous antioxidant responses. During cell peroxidation, Keap1 expression was inhibited in the cytoplasm, and NRF2-Keap1 dissociated, which promoted the entrance of NRF2 into the nucleus, upregulated the production of antioxidant proteins, and reduced levels of ROS and oxidative stress injury [39, 40].

The NRF2 protein was continuously degraded by Keap1; therefore, downregulation of Keap1 expression could promote NRF2 expression in the nucleus and increase levels of antioxidant proteins [41]. Activation of the P62-Keap1-NRF2 pathway was negatively correlated with ferroptosis in hepatoma cells. Knockdown of P62 expression resulted in upregulation of Keap1 expression, which enhanced the degradation of NRF2 and decreased the entry of NRF2 into the nucleus; the antioxidant proteins such as HO1, FTH1, and NQO1 were decreased and thus enhance the sensitivity of drug-induced ferroptosis in hepatoma cells [23].

This study demonstrated that the expression of P62 and NRF2, as well as the levels of HO1, FTH1, and NQO1, was increased in RILI cells, and Keap1 was downregulated, while the overall change was insignificant. A possible explanation is the activation of the P62-Keap1-NRF2 signaling pathway to stabilize redox balance in RILI cells in response to peroxide exposure. Additionally, Fer-1 treatment did not induce P62 protein and mRNA expression in RILI cells, while it significantly induced Keap1 protein expression, but not its mRNA level. Both NRF2 protein and mRNA levels increased significantly, as did levels of antioxidant proteins HO1, FTH1, and NQO1. In summary, our results suggest that Fer-1 activates NRF2 signaling pathway, increases antioxidant protein, reduces ROS production, and thus inhibits ferroptosis. Fer-1 may act at NRF2, rather than P62 and Keap1. It is possible that the decrease of transcription-dependent of Keap1 may be caused by the activation of NRF2 by Fer-1, the enhanced Keap1-NRF2 complex, and the continuous degradation of Keap1.

To further investigate the role of P62-Keap1-NRF2 in regulating ferroptosis in RILI cells, we constructed plasmids expressing P62 and NRF2 and siRNAs targeting Keap1 into RILI cells. Our results showed that upregulation of P62 contributed to downregulation of Keap1 and promoted NRF2 to transfer into nucleus, and thus, the levels of antioxidant proteins HO1, FTH1, and NQO1 in RILI cells as well as GSH and Fe^2+^ were increased, while the levels of ROS and MDA were decreased. Additionally, the number of intracellular shrinkage and membrane low potential mitochondria decreased, the level of GPX4 increased, and the level of ACSl4 decreased, indicating that the intracellular ferroptosis was inhibited. These results confirm that activating the P62-Keap1-NRF2 signaling pathway inhibits ferroptosis in RILI.

## 5. Conclusions

**Figure 9:**
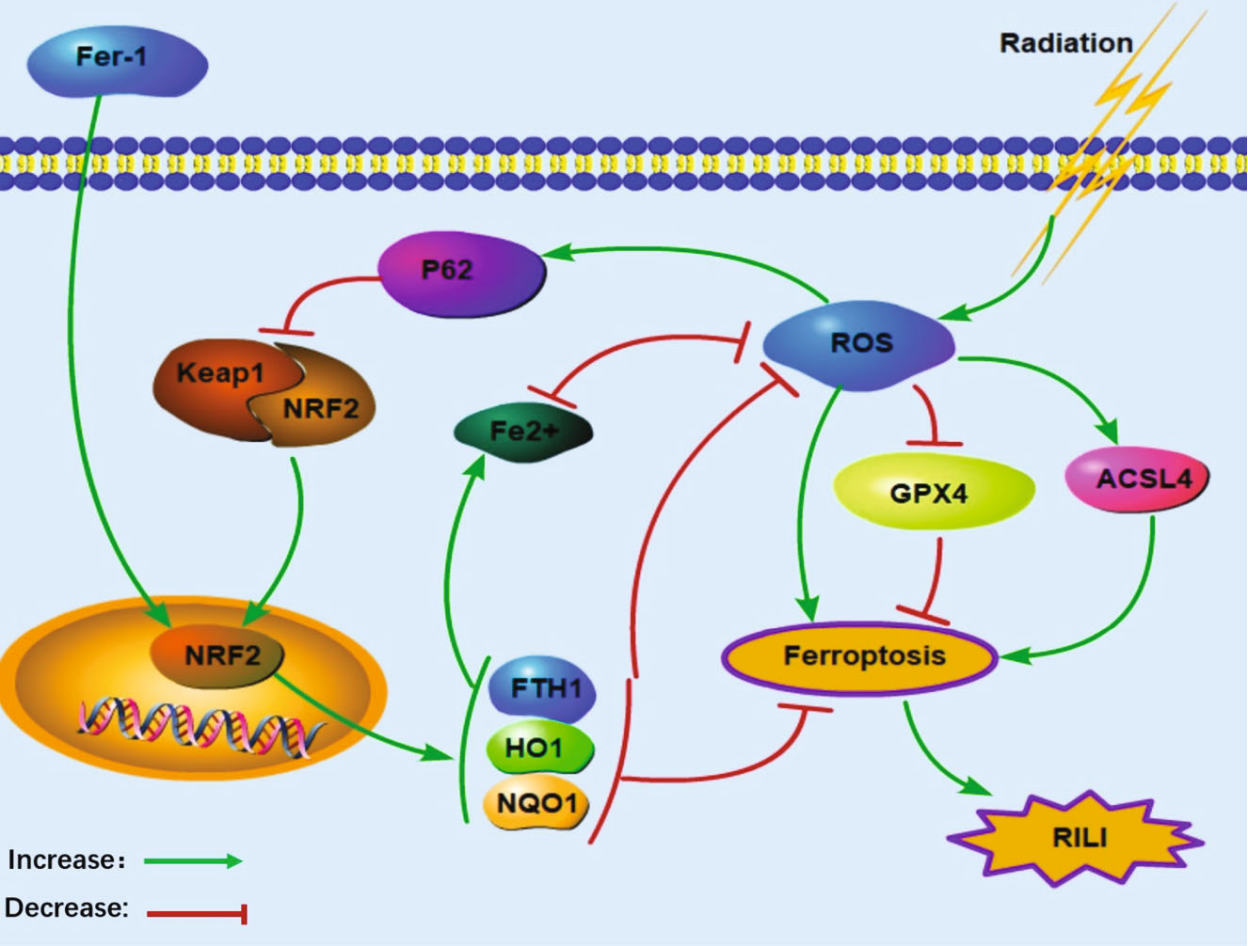
The schematic diagram of P62-Keap1-NRF2 pathway following RILI.

In summary, we successfully established the RILI cell model and demonstrated a form of regulated cell death, ferroptosis, caused by irradiation, which differs from the classical cell apoptosis. Ferroptosis interference may open a new path to reduce ROS damage and protect against RILI. In RILI, as shown in Figure 9, a large number of ROS are generated by radiation ionization. During the oxidative stress state, the P62-Keap1-NRF2 antioxidant signaling pathway was activated, but the increase in NRF2 expression was limited. Fer-1 can activate the NRF2 signaling pathway in RILI, leading to upregulating antioxidant protein expression, reducing ROS production and preventing ferroptosis. P62-Keap1-NRF2 signaling pathway is involved in the regulation of ferroptosis in RILI. By activating P62, Keap1 expression was blocked, which converted NRF2 into nuclear for expression, and NRF2 antioxidant signaling pathway is activated, which yields an increase in antioxidant protein production and a decrease in ROS level, and finally inhibited ferroptosis. The activation of the P62-Keap1-NRF2 signaling pathway represents a potential therapeutic approach to prevent RILI.

## Data Availability

All data generated or analyzed during this study are included in this published article.

## Abbreviations

RILI: Radiation-induced lung injury
RIP: Radiation-induced pneumonitis
RILF: Radiation-induced lung fibrosis
RCD: Regulated cell death
ROS: Reactive oxygen species
Keap1: Kelch-like ECH-associated protein 1
NRF2: Nuclear factor erythroid 2-related factor 2
Fer-1: Ferrostatin-1
HO1: Heme oxygenase 1
NQO1: Quinone oxidoreductase 1
FTH1: Ferritin heavy chain 1
PMSF: Phenylmethylsulfonyl fluoride
PVDF: Polyvinylidene fluoride
TEM: Transmission electron microscopy
MDA: Malondialdehyde
GSH: Glutathione
GPX4: Glutathione peroxidase 4
ACSL4: Acyl-CoA synthetase long-chain family member 4
IR: Irradiation.
